# Mechanobiological and microenvironmental roles of lymph nodes in cancer: implications for lymph node de-escalation

**DOI:** 10.3389/fmed.2026.1870790

**Published:** 2026-07-07

**Authors:** Xiaoling Chen, Mengting Jiang, Xiaolong Liu, Xiaoxiao Fan, Guoqiao Chen

**Affiliations:** 1 Department of Nursing, Sir Run Run Shaw Hospital, Zhejiang University School of Medicine, Hangzhou, China; 2 Department of General Surgery, Sir Run Run Shaw Hospital, School of Medicine, Zhejiang University, Hangzhou, China

**Keywords:** de-escalation, immunosuppression, lymph node, tumor mechanobiology, tumor microenvironment

## Abstract

Prophylactic lymph node (LN) dissection has long been regarded as an important component of surgical treatment for many solid tumors, based largely on the traditional anatomical view that LNs serve as “relay stations” for tumor metastasis. However, recent clinical studies in melanoma, breast cancer, and other malignancies have increasingly suggested that, in patients with clinically negative or low-burden positive LNs, routine extensive LN dissection does not always translate into a clear overall survival benefit and may instead increase complications such as lymphedema and neurovascular injury. In parallel, advances in tumor mechanobiology have provided a new theoretical framework for reinterpreting lymphatic metastasis and LN management strategies. Increased extracellular matrix stiffness, accumulation of solid stress, and elevated interstitial fluid pressure within the tumor microenvironment (TME) together constitute a coupled solid stress-fluid stress system. This system not only promotes tumor cell invasion, lymphangiogenesis, and directed migration toward the lymphatic system, but also regulates the functions of dendritic cells, macrophages, lymphatic endothelial cells (LEC), and T cells, thereby driving the formation of an immunosuppressive premetastatic niche in tumor-draining LNs (TDLNs). From this perspective, LNs should be understood not merely as anatomical structures, but as functional hubs of tumor-immune-mechanical interaction. Based on current evidence from melanoma, breast cancer, head and neck squamous cell carcinoma, lung cancer, and gastrointestinal malignancies, this article discusses the theoretical basis, applicable boundaries, and translational significance of LN de-escalation strategies, and reviews the dual role of TDLNs in metastasis and antitumor immunity from the intersecting perspectives of tumor biomechanics, lymphatic biology, and tumor immunology. We propose that LN management should shift from a traditional anatomy-oriented approach to a function-oriented precision strategy, thereby providing a new theoretical basis for future cancer treatment.

## 1 Introduction

The evolution of lymph node (LN) management reflects a broader shift in oncologic surgery from “maximal resection” toward “maximal benefit with minimal harm.” For a long time, regional LNs were regarded as a sequential route of tumor spread, and prophylactic or completion LN dissection was therefore widely incorporated into routine surgical practice. Although this strategy has clear value for staging, local control, and postoperative treatment planning, advances in sentinel LN biopsy (SLNB), imaging-based risk stratification, and systemic therapy have increasingly challenged the practice of routine extended dissection based solely on the “transit station” hypothesis (1, 2).

Current evidence suggests that LNs are not merely passive containers for metastatic deposits during tumor progression, but rather dynamic organs with dual roles in immune activation and immune tolerance. On the one hand, tumor-draining LNs (TDLN) serve as key sites for antigen presentation, initial T-cell priming, and responses to immune checkpoint blockade (ICB). On the other hand, tumor-associated alterations in lymphatic flow, lymphatic endothelial cell (LEC) remodeling, and the accumulation of macrophages and regulatory T cells (Treg) may progressively convert them into a premetastatic niche that favors tumor cell seeding and immune evasion (1, 3). Reframing the role of LNs in cancer treatment is therefore a prerequisite for evaluating whether “de-escalation” is truly justified.

In recent years, the rise of tumor mechanobiological research has provided a deeper explanatory framework for this issue. Unlike traditional approaches that focus primarily on molecular signaling, mechanobiology emphasizes the systemic influence of physical factors such as extracellular matrix (ECM) stiffness, solid stress (SS), interstitial fluid pressure (IFP), fluid shear forces, and pressure gradients on tumor cell behavior, lymphatic drainage, and immune responses. This perspective implies that the decision to dissect LNs should not be determined solely by anatomical positivity or negativity, but should also take into account whether, at a given time point, the LN is functioning predominantly as a “metastasis-promoting node” or an “immune-response node.”

LNs are highly organized secondary lymphoid organs that function as central hubs connecting peripheral tissues with the adaptive immune system (4, 5). Structurally, lymph enters the LN through afferent lymphatic vessels and first reaches the subcapsular sinus before passing through cortical, paracortical, and medullary compartments (5). Within this microanatomical network, dendritic cells (DCs) transport tumor-associated antigens from peripheral tissues and present them to naïve T cells, thereby initiating adaptive immune responses (4). Fibroblastic reticular cells (FRCs), lymphatic endothelial cells (LECs), and high endothelial venules (HEVs) collectively regulate immune-cell trafficking, antigen transport, and lymphocyte activation (5). In the context of cancer, TDLNs serve not only as the first regional sites exposed to tumor-derived antigens and soluble factors, but also as critical regulators of both antitumor immunity and metastatic dissemination (1). Therefore, understanding the structural organization and functional plasticity of LNs provides an important foundation for interpreting how tumor-associated mechanical cues and lymphatic remodeling influence LN biology and the rationale for LN de-escalation strategies.

## 2 LN de-escalation in current tumor

**Figure 1:**
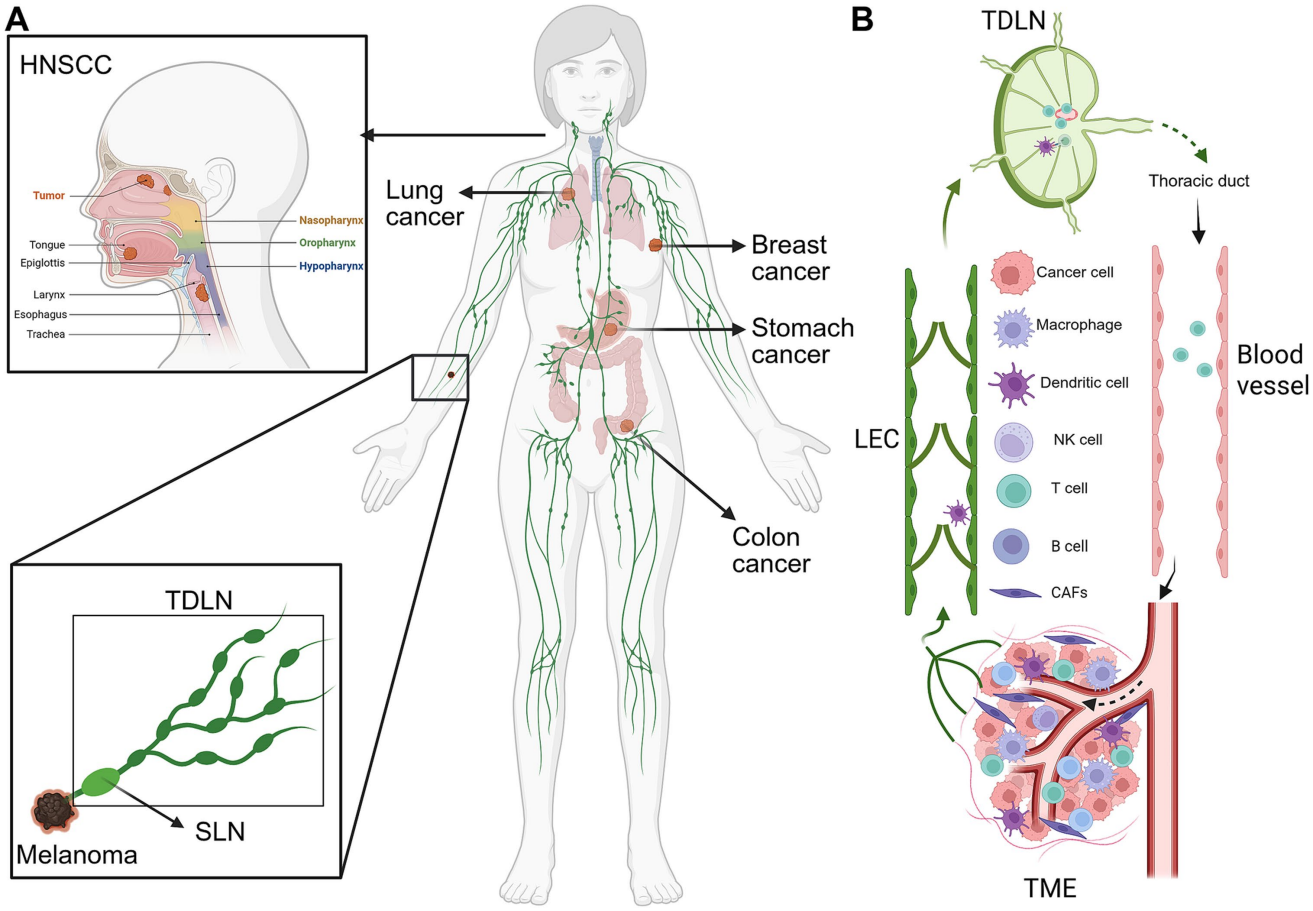
Lymph node distribution and lymphatic drainage across major solid tumors. **(A)** Representative lymphatic drainage pathways and tumor-draining lymph node (TDLN) basins in major solid tumors, including melanoma, breast cancer, head and neck squamous cell carcinoma (HNSCC), lung cancer, stomach cancer, and colon cancer. Sentinel lymph nodes (SLNs) represent the first draining lymph nodes receiving lymphatic flow from the primary tumor and constitute the primary targets for nodal staging and de-escalation strategies. **(B)** Schematic illustration of lymphatic transport between the tumor microenvironment (TME) and TDLNs. Tumor-associated antigens, soluble factors, extracellular vesicles, and antigen-presenting cells are transported through lymphatic vessels lined by lymphatic endothelial cells (LECs) toward draining lymph nodes. Within TDLNs, dendritic cells present antigens to naïve T cells, initiating antitumor immune responses. Activated lymphocytes subsequently re-enter systemic circulation through efferent lymphatic vessels and the thoracic duct. The figure highlights the dual role of TDLNs as sites of both immune activation and metastatic dissemination. HNSCC, head and neck squamous cell carcinoma; TDLN, tumor-draining lymph node; SLN, sentinel lymph node; LEC, lymphatic endothelial cell; TME, tumor microenvironment. Created in BioRender. Chen, G. (2026) https://BioRender.com/n7sbscw.

The concept of de-escalation in cancer therapy has moved beyond the traditional “more is better” paradigm, with the goal of reducing treatment-related adverse events, preserving quality of life, and maintaining oncologic outcomes in appropriately selected patients (6). So-called LN de-escalation is not simply a reduction in the extent of surgery. Rather, it refers to minimizing unnecessary dissection, irradiation, or overall treatment intensity while preserving oncologic control and staging accuracy, thereby reducing complications and functional impairment (7–9). This approach is based on the recognition that not every pathologically positive LN represents an obligatory linear step toward distant metastasis, and not every clinically node-negative patient derives benefit from prophylactic nodal dissection (10, 11). At the same time, TDLNs with the sentinel LN (SLN) representing the first draining node, are central to both nodal metastasis and antitumor immune responses. On this basis, we review the evolution of clinical LN de-escalation strategies in breast cancer (BC), melanoma (MM) and head and neck squamous cell carcinoma (HNSCC), and further discuss their potential relevance in lung cancer (LC), stomach cancer (SC) and colon cancer (CC) (Figure 1A; Table 1).

**Table 1:** De-escalation trends in lymph node management across major solid tumors.

Cancer type	Current guideline approach	De-escalation trend	Key supporting evidence
Breast cancer	SLNB is standard; SLNB may be omitted in selected low-risk patients; ALND can be avoided in patients with limited SLN involvement	Well established	ACOSOG Z0011 (10): SLNB alone is non-inferior to ALND in patients with 1–2 positive SLNs; AMAROS (25): axillary radiotherapy provides comparable control with less morbidity; SOUND/INSEMA (24): omission of SLNB is feasible in selected low-risk patients
Melanoma	SLNB is used for staging; CLND is no longer routinely recommended after positive SLN	Well established	MSLT-II (11): CLND improves regional control but not survival; DeCOG-SLT (23): no survival benefit with CLND, supporting observation after SLN positivity
NSCLC	Systematic mediastinal lymph node evaluation (dissection or sampling) is recommended; SLNB is not standard	Controversial not practice-changing	ACOSOG Z0030 (28): no survival benefit of complete mediastinal dissection over systematic sampling in early-stage patients with negative nodes
HNSCC	Cervical lymph node management remains essential; elective neck dissection or SLNB is used in early-stage disease; regional nodal irradiation is common	Limited mainly in OSCC	D’Cruz et al., NEJM 2015 (29): elective neck dissection improves survival compared with observation; ongoing trials (NRG-HN006 (28)) are evaluating SLNB as a less invasive alternative
Colon cancer	Radical resection with adequate lymph node retrieval (≥12 nodes) remains standard	No clear de-escalation trends	Observational studies (90) consistently show that adequate lymph node retrieval is associated with accurate staging and improved outcomes; no randomized evidence supports reducing nodal evaluation
Stomach cancer	Radical gastrectomy with D2 lymphadenectomy remains standard, especially in East Asia	No true de-escalation	Dutch D1/D2 trial (15-year follow-up) (91): D2 reduces locoregional recurrence and disease-specific mortality; JCOG9501: extended para-aortic dissection beyond D2 provides no survival benefit

SLNB, sentinel lymph node biopsy; SLN, sentinel lymph node; ALND, axillary lymph node dissection; CLND, completion lymph node dissection; NSCLC, non-small cell lung cancer; HNSCC, head and neck squamous cell carcinoma; OSCC, oral squamous cell carcinoma.

### 2.1 Breast and melanoma cancer

BC and MM have shown the clearest clinical trend toward LN de-escalation. According to the traditional Halsted theory, tumor spread was considered to occur in a stepwise manner through the lymphatic system. On this basis, complete dissection of TDLN was believed to interrupt metastatic spread and improve the chance of cure, and the favorable outcomes observed in a subset of patients with BC further reinforced this concept (12, 13). Following this precedent, increasingly extensive procedures, including radical mastectomy, were advocated in an effort to reduce the risk of metastasis (14). The principle of complete lymph node dissection (CLND) was subsequently extended to other malignancies, such as MM (15), LC (16), SC (17), and CC (18). However, clinical observations, including early experience in penile cancer, together with the development of lymphatic mapping, suggested that limited nodal surgery might reduce treatment-related morbidity such as lymphedema while still identifying patients at highest risk of occult nodal metastasis (19, 20). Because the SLN is the first draining node from the primary tumor and harbors metastatic involvement in a substantial proportion of node-positive cases, it gradually became the key target for nodal staging. This shift provided the conceptual basis for preserving non-sentinel TDLNs and supported the development of LN de-escalation strategies in both BC (20) and SC (21).

More importantly, evidence from large perspective trials, together with advances in preoperative staging techniques such as SLNB, has established LN de-escalation as a generally accepted approach in selected patients with BC and MM. In MM, the MSLT-II and DeCOG-SLT trials consistently showed that although CLND improves regional disease control, it does not provide a survival benefit (22, 23). In BC, trials such as ACOSOG Z0011, AMAROS, and SOUND demonstrated that reduced axillary intervention can be safely implemented in appropriately selected patients without compromising survival outcomes (10, 24, 25). Current guidelines further support this trend. In early BC, SLNB may be omitted in selected low-risk patients, and axillary lymph node dissection (ALND) is not recommended for patients with clinically node-negative disease, one or two positive SLNs, breast-conserving surgery, and whole-breast irradiation (26). Likewise, the ESMO 2024 MM guideline recommends that the indication for SLNB should be determined according to Breslow thickness, ulceration status, and other risk factors. SLNB is not routinely recommended for pT1a MM, defined as a tumor thickness of <0.8 mm without ulceration. In addition, routine CLND is no longer recommended after a positive SLN (27).

### 2.2 Lung and head and neck cancers

In lung cancer, particularly non-small cell lung cancer (NSCLC), mediastinal lymph nodes (MLNs) constitute the principal tumor-draining nodal basin, whereas in HNSCC the cervical lymph nodes (CLNs) serve as the major draining nodes. Compared with BC and MM, however, evidence supporting LN de-escalation in lung and HNSCC remains less mature. In both settings, CLND is still performed primarily for accurate pathological staging and treatment planning. In NSCLC, the ACOSOG Z0030 trial showed that, after rigorous systematic nodal sampling, complete MLN dissection did not improve survival compared with sampling alone (28). By contrast, in early oral squamous cell carcinoma (OSCC), elective neck dissection was associated with better survival than therapeutic neck dissection performed only after nodal relapse (29). Although SLNB is well established in MM and BC, it has not been widely adopted in LC because of the complex pulmonary lymphatic drainage pattern, the frequent occurrence of skip metastasis, and the continued need for precise nodal staging. Accordingly, current guidelines still recommend systematic nodal dissection or sampling rather than SLNB (30). In HNSCC, nodal de-escalation has been explored mainly in early-stage clinically node-negative OSCC, where SLNB is being evaluated as an alternative to elective neck dissection, but robust evidence supporting broad omission of neck dissection remains limited, and current efforts are driven primarily by morbidity reduction (31).

### 2.3 Stomach and colon cancers

In SC, LN de-escalation has been investigated mainly in early gastric cancer through SLN navigation surgery, but current evidence remains insufficient to support its routine adoption. The JCOG0302 trial identified a clinically relevant false-negative risk in intraoperative sentinel node assessment, and the phase III SENORITA trial showed that although stomach-preserving surgery improved postoperative quality of life, it did not demonstrate noninferior 3-year disease-free survival compared with standard gastrectomy plus lymphadenectomy (32–35). In CC, evidence for nodal de-escalation is even weaker: although SLN mapping and ultra-staging may improve the detection of occult nodal disease, these approaches have not replaced standard colectomy with conventional lymphadenectomy because of variable lymphatic drainage, the possibility of skip or aberrant nodal spread, and the continued recommendations of current guidelines (36–40).

Clinical evidences supporting LN de-escalation varies across tumor types and largely depends on differences in patient prognosis and disease biology. In the era of systemic cancer therapy, particularly with the rapid development of immunotherapy, the role of metastatic LN can no longer be viewed solely from a surgical perspective. LN metastasis may not be merely a passive process of tumor spread, but is closely associated with the tumor microenvironment (TME) remodeling, immune regulation, and multiple biomechanical factors (3, 41). In this context, mechanobiological research, spanning theoretical, mechanistic, and preclinical studies, may provide an important foundation for understanding and advancing LN de-escalation.

## 3 Mechanobiology of TME and LN immunosensors

TDLNs function as critical immunological sensors that integrate signals from the solid tumor via lymphatic drainage. Antigen-presenting cells, particularly DCs, capture tumor-associated antigens in the tumor microenvironment and subsequently migrate along lymphatic vessels toward TDLNs. This process is facilitated by lymphatic flow as well as chemokine gradients, such as the CCR7–CCL21 axis, and is often accompanied by tumor-induced lymphangiogenesis. Upon reaching the LNs, DCs present antigens to naïve T cells, thereby initiating T cell priming and activation (3, 4, 42). Activated T cells then exit the LNs through efferent lymphatics, enter the systemic circulation via the thoracic duct, and eventually home to the tumor TME, where they exert anti-tumor immune functions (5, 43) (Figure 1B). Notably, the interaction between TDLNs and the TME represents a dynamic and context-dependent balance rather than a simple linear cascade. On one hand, TDLNs can promote effective anti-tumor immunity through T cell priming. On the other hand, tumor-derived factors can reprogram TDLNs into an immunosuppressive niche. This interplay can be conceptualized as a “tug-of-war” between immune activation and immune tolerance. The dominance of either TME-driven immunosuppression or TDLN-mediated immune activation ultimately determines disease progression or control (44, 45). In the following section, we summarize the mechanobiological characteristics of both the TME and TDLNs, highlighting how mechanical cues and lymphatic flow contribute to this immunological balance.

### 3.1 How aberrant tumor mechanobiology drives lymphatic metastasis

**Figure 2:**
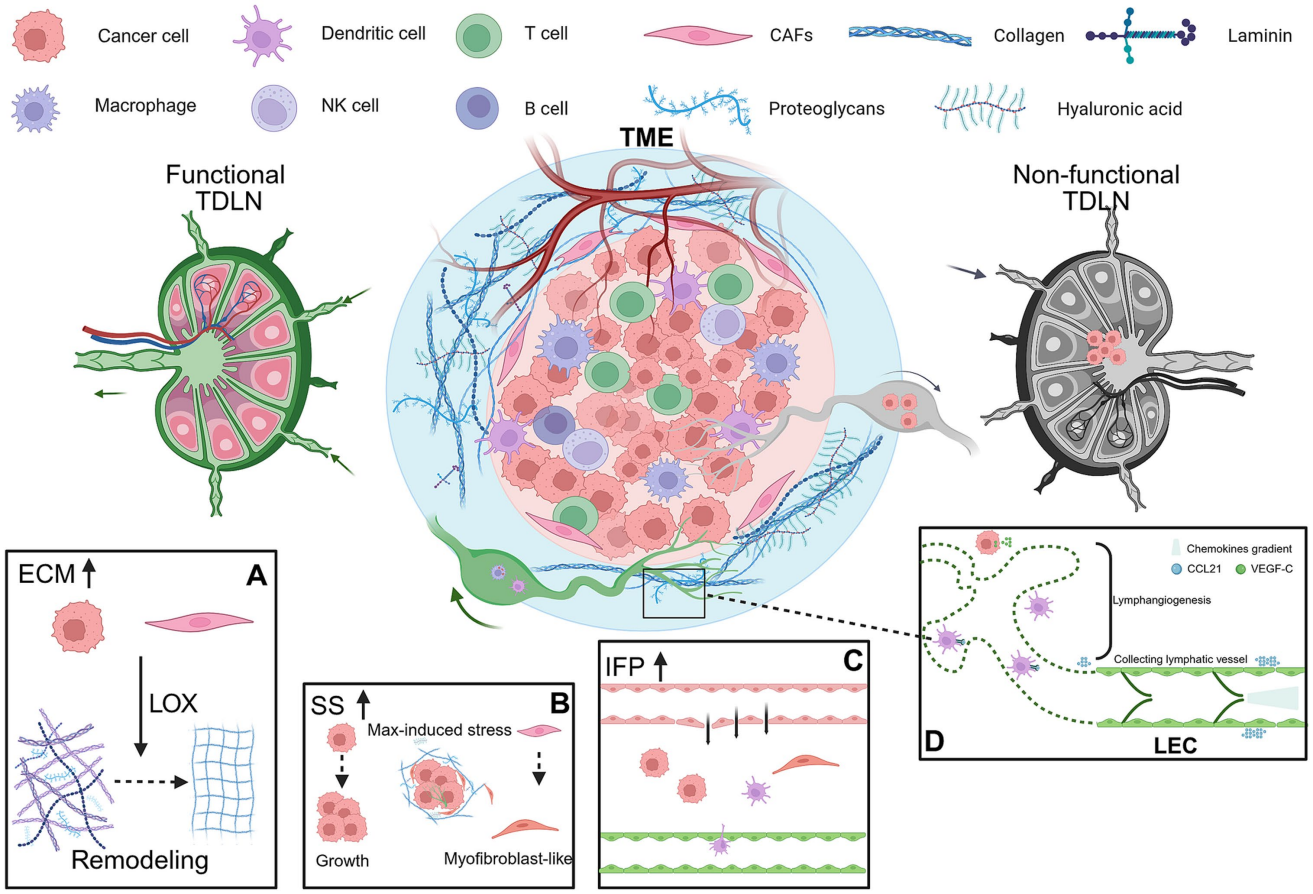
Mechanobiological features of the tumor microenvironment. The tumor microenvironment (TME) contains multiple interacting mechanical components, including extracellular matrix (ECM) remodeling, solid stress (SS), and elevated interstitial fluid pressure (IFP). Together, these biomechanical abnormalities reshape lymphatic drainage, regulate immune-cell trafficking, and facilitate the transport of tumor-derived antigens, extracellular vesicles, cytokines, and chemokines to tumor-draining lymph nodes (TDLNs). Depending on the balance between immune activation and immune suppression, TDLNs may remain functionally immune-reactive or become remodeled into immunosuppressive and metastasis-permissive niches. The figure summarizes how mechanical abnormalities within the TME influence lymphatic dissemination, immune regulation, and lymph node function. **(A)** Increased collagen deposition, lysyl oxidase (LOX)-mediated crosslinking, and cancer-associated fibroblast (CAF)-driven matrix remodeling promote ECM stiffening, facilitate tumor-cell invasion, and alter immune-cell migration within the TME. **(B)** Progressive tumor growth within a confined tissue space, together with cancer-associated fibroblast (CAF)-mediated matrix contraction and myofibroblast-like activation, generates and accumulates solid stress (SS). This stored mechanical stress deforms the surrounding tissue architecture, compresses blood and lymphatic vessels, and contributes to hypoxia and abnormal stromal remodeling. **(C)** Tumor-associated angiogenesis produces structurally abnormal and hyperpermeable blood vessels, leading to fluid leakage into the interstitial space and elevation of interstitial fluid pressure (IFP). The resulting pressure gradients generate interstitial fluid flow toward functional peripheral lymphatic vessels, promoting the transport of soluble factors and the formation of chemokine gradients that guide tumor-cell migration, immune-cell trafficking, and lymphatic dissemination. **(D)** Tumor-derived VEGF-C promotes lymphatic remodeling and expansion of functional peritumoral lymphatic vessels. Concurrently, lymphatic endothelial cells (LECs) produce chemokines such as CCL21, establishing gradients that guide the migration of CCR7-expressing tumor cells and dendritic cells toward lymphatic vessels and tumor-draining lymph nodes (TDLNs). These processes facilitate both lymphatic dissemination of tumor cells and antigen transport to TDLNs, thereby contributing to the dynamic balance between metastatic progression and antitumor immune activation. ECM, extracellular matrix; CAF, cancer-associated fibroblast; LOX, lysyl oxidase; SS, solid stress; IFP, interstitial fluid pressure; VEGF-C, vascular endothelial growth factor-C; TDLN, tumor-draining lymph node; LEC, lymphatic endothelial cell. Created in BioRender. Chen, G. (2026) https://BioRender.com/058hvqr.

Lymphatic metastasis is not merely a consequence of passive tumor cell entry into pre-existing lymphatic vessels. Instead, it is shaped by a constellation of mechanical abnormalities within the TME, including ECM stiffness, SS, and IFP. These physical cues interact with biochemical signaling to regulate tumor cell migration, invasion, lymphatic entry, and premetastatic LN remodeling (Figure 2).

#### 3.1.1 Extracellular matrix stiffness

ECM stiffness is one of the most extensively studied mechanical abnormalities in solid tumors. It is comprised with cancer-associated fibroblast (CAF), proteoglycans, collagen, laminin, and hyaluronic acid (HA), and creates mechanical tracks for invasion (Figure 2). During tumor progression, increased collagen deposition, lysyl oxidase (LOX)-mediated collagen crosslinking, and CAF-driven matrix remodeling collectively stiffen the tumor stroma. This stiffened ECM enhances tumor cell contractility, focal adhesion formation, integrin signaling, and directional invasion.

Paszek et al. used three-dimensional (3D) mammary epithelial culture models and mechanically tunable matrices to show that increased matrix stiffness promotes a malignant phenotype by enhancing integrin clustering, focal adhesion assembly, ERK activation, and Rho-dependent cytoskeletal tension (46). Levental et al. further provided direct experimental evidence linking ECM stiffening to tumor progression (47). Using breast cancer models, collagen crosslinking manipulation, and integrin signaling analysis, they demonstrated that LOX-mediated collagen crosslinking stiffens the ECM, promotes focal adhesion formation, enhances PI3K signaling, and induces invasion of oncogene-initiated mammary epithelium. This study established a causal relationship between matrix stiffness, integrin mechanotransduction, and invasive behavior. Beyond stiffness magnitude, ECM architecture also determines invasion direction. Provenzano et al. used mammary tumor models and second harmonic generation imaging to characterize tumor-associated collagen signatures (TACS) (48). They found that collagen fibers at the tumor-stromal interface progressively reorganize from dense peritumoral deposition to radially aligned fibers, along which tumor cells preferentially invade. In tumor explants embedded in collagen matrices, tumor cells were observed to migrate outward along these aligned collagen fibers, suggesting that ECM remodeling provides physical guidance tracks for local invasion and potentially for subsequent entry into lymphatic vessels.

Taken together, these studies suggest that ECM stiffness and collagen alignment do not simply reflect tumor desmoplasia. Rather, they actively convert the tumor margin into a mechanically permissive interface that promotes directional invasion toward stromal and lymphatic routes.

#### 3.1.2 Solid stress

SS refers to mechanical stress stored and transmitted by the solid components of tumors, including cancer cells, stromal cells, and ECM. SS compresses vessels and reshaping the metastatic microenvironment, and it arises from uncontrolled tumor growth in a confined space and from stromal contraction and matrix remodeling. SS can compress blood and lymphatic vessels, impair perfusion, promote hypoxia, and indirectly enhance invasive and metastatic behavior.

Stylianopoulos et al. developed mathematical models of tumor growth and combined them with *in vivo* experiments to distinguish growth-induced SS from fluid pressure (49). They showed that SS accumulates during tumor growth and is related to tumor volume in a nonlinear manner. Histological analysis further demonstrated that SS can deform vessels in and around tumors, whereas IFP alone is insufficient to explain vessel compression. This finding is important because vessel collapse contributes to hypoxia and abnormal transport, both of which can promote invasive phenotypes and reduce treatment delivery. Besides, Chauhan et al. provided additional experimental evidence in pancreatic tumor models (50). They showed that hyaluronan accumulation contributes to SS and compresses tumor blood vessels. By depleting HA, vascular compression was alleviated and perfusion improved, demonstrating that stromal solid components can mechanically regulate vascular function. Although this study focused mainly on drug delivery and perfusion, its implication for lymphatic metastasis is important: compressed vascular and lymphatic networks can alter fluid drainage routes, reinforce pressure gradients, and create hypoxic niches that favor invasion.

SS may therefore promote lymphatic dissemination indirectly by altering tumor perfusion, increasing hypoxia-driven invasive signaling, and changing the routes through which interstitial fluid and tumor-derived material drain toward lymphatics. In this sense, SS acts as an upstream mechanical organizer that reshapes the physical boundary conditions of the TME.

#### 3.1.3 Interstitial fluid pressure

IFP is another hallmark of solid tumors. Leaky tumor blood vessels, impaired lymphatic drainage, and dense ECM collectively elevate IFP. IFP generates outward transport toward lymphatic vessels. Because IFP is often high throughout the tumor core but drops sharply at the tumor margin, it generates outward pressure gradients that drive convective interstitial flow toward surrounding stromal tissue and lymphatic vessels.

Stylianopoulos et al. showed that tumor fluid pressure increases during tumor progression due to vascular hyperpermeability and impaired lymphatic drainage (49). The functional importance of IFP-driven transport was demonstrated by studies of interstitial flow and tumor cell migration. Polacheck et al. designed a microfluidic 3D collagen culture system that allowed stable pressure gradients and direct visualization of tumor cell migration under controlled interstitial flow (51). They found that interstitial flow biases the direction of tumor cell migration in a flow-rate- and cell-density-dependent manner. Blocking CCR7 signaling altered migration in the flow direction, supporting a model in which pressure-driven flow shapes autologous chemokine gradients that guide tumor cell movement. Moreover, Shields et al. earlier proposed the concept of autologous chemotaxis (52). Using 3D matrix models, they showed that interstitial flow caused by lymphatic drainage can direct tumor cell migration through autocrine CCR7 signaling. In their model, flow redistributed tumor-secreted chemokines such as CCL21 around tumor cells, generating local gradients that promoted migration toward lymphatic vessels. This provided a mechanistic explanation for how elevated IFP and lymphatic drainage can bias tumor cells toward lymphatic routes.

Thus, IFP can serve as a physical driver of lymphatic dissemination by generating outward convective transport and chemokine gradient formation at the tumor margin.

#### 3.1.4 Spatial zonal lymphatic drainage

Various mechanical factors within the TME critically regulate lymphatic drainage and thereby influence tumor dissemination. Although functional lymphatic drainage is thought to occur predominantly through peritumoral lymphatic vessels, intratumoral lymphatic structures may represent a dynamic outcome of tumor expansion rather than a pre-existing independent drainage compartment (53, 54). During early tumor growth, lymphatic vessels located at the tumor–stromal interface may initially function as peritumoral lymphatics (49, 55, 56). As the tumor mass expands, however, progressive ECM deposition, increased SS, elevated IFP, and stromal contraction may mechanically incorporate, deform, or compress these vessels, converting them into intratumoral or functionally impaired lymphatic structures. This interpretation is consistent with experimental evidence showing that proliferating cancer cells and tumor-associated SS can compress intratumoral vessels, including fragile lymphatic vessels, whereas functional lymphatic drainage is mainly preserved at the tumor margin (51, 55). Padera et al. demonstrated in mouse tumor models that VEGF-C increased lymphatic density at the tumor margin and promoted LN metastasis, while functional lymphatics remained absent within the tumor core (53).

This spatial transition may have immunological consequences. The tumor core is frequently characterized by high mechanical compression, hypoxia, necrosis, elevated IFP, and accumulation of immunosuppressive mediators such as TGF-*β* (57–59). These conditions may increase the release of apoptotic or necrotic tumor debris, extracellular vesicles (EV), soluble tumor antigens, and cytokines (59, 60). Even if intratumoral lymphatic vessels are poorly functional as drainage conduits, tumor-derived materials from the mechanically stressed core may be transported outward by pressure-driven interstitial flow and eventually enter functional peritumoral lymphatics (51, 52, 61). Thus, the biological content delivered to TDLN may reflect not only signals generated at the tumor margin but also the integrated output of compressed and immunosuppressive tumor-core regions.

A recent mechanistic study further supports the possibility that tumor debris delivered to TDLNs can actively induce immune tolerance (62). Lamorte et al. showed that, after cancer therapy, medullary sinus macrophages (MSMs) in TDLNs avidly phagocytose dying tumor cell debris and acquire a tolerogenic phenotype. Mechanistically, efferocytosis of tumor debris by MSMs induced IL-33 expression, which activated ST2-expressing regulatory T cells in TDLNs. These Treg cells then migrated back to the tumor and suppressed CD8 + T cell-mediated anti-tumor immunity. Genetic deletion of IL-33 in MSMs or blockade of IL-33 receptor ST2 enhanced therapeutic responses, demonstrating that the MSM–IL-33/ST2–Treg axis can convert tumor-derived debris into systemic immune suppression.

Based on these findings, we propose a spatial mechanics–immune fate hypothesis: as tumors grow, peritumoral lymphatics may be progressively incorporated into the tumor mass and become compressed intratumoral lymphatic structures, while functionally active peritumoral lymphatics remain the major exit routes for lymphatic drainage. Mechanical stress within the tumor core may increase the generation of tumor debris and immunosuppressive signals, which are transported to TDLNs through residual or peripheral lymphatic routes. Once in the LN, these materials may be processed by MSMs and induce IL-33/ST2-dependent Treg activation, thereby promoting immune tolerance and tumor progression. However, this model remains hypothetical and should be tested by spatial lymphatic tracing, intratumoral regional tracer injection, single-cell profiling of TDLNs, and paired analysis of metastatic and non-metastatic LNs.

Although this hypothesis is proposed as a general framework linking tumor mechanics, lymphatic transport, and immune regulation, the relative contribution of mechanobiological mechanisms—including ECM remodeling, SS, elevated IFP, and lymphatic reprogramming—may differ substantially among tumor types. BC is characterized by pronounced ECM remodeling, collagen deposition, and LOX-mediated matrix stiffening, which have been closely linked to tumor invasion and metastatic progression (47, 59). In contrast, MM generally exhibits a less desmoplastic stromal architecture but displays strong lymphangiogenic activity, and lymphatic remodeling has been implicated as a major driver of LN dissemination (44). In HNSCC, chronic inflammation, extensive lymphatic network remodeling, and immune suppression within TDLNs are prominent features associated with regional metastasis (63). LC exhibit considerable heterogeneity in stromal composition and lymphatic drainage patterns, whereas gastrointestinal malignancies, particularly SC, often demonstrate abundant stromal fibrosis and complex regional lymphatic networks that may influence both metastatic spread and nodal immune regulation. These observations suggest that the mechanobiological determinants of lymphatic dissemination and TDLN remodeling are likely tumor-specific. Consequently, the applicability and biological rationale of LN de-escalation strategies may vary across different cancer types and should be interpreted within the context of their distinct mechanical and immune microenvironments.

Collectively, these observations suggest that mechanical abnormalities within the TME influence not only tumor-cell dissemination but also lymphatic transport and immune regulation within TDLNs. Despite tumor-specific differences, several common mechanobiological pathways appear to govern the communication between primary tumors and draining LNs. To facilitate integration of these concepts, the major mechanobiological factors discussed in this section and their potential effects on lymphatic dissemination and TDLN immunity are summarized in Table 2.

**Table 2:** Key mechanobiological features of the TME and their effects on TDLNs.

Factors	Representative molecular changes	Effect on lymphatic system	Effect on TDLN immunity	Predominant consequence
ECM stiffness	Collagen I, LOX, Integrin β1	Enhanced invasion	Increased transport of tumor-derived factors	Mixed
Solid stress	HA, HIF-1α, TGF-β	Vessel compression	Immunosuppressive niche formation	Immune suppression
Elevated IFP	CCR7, CCL21	Interstitial flow generation	Enhanced tumor–TDLN communication	Mixed
Spatial lymphatic drainage	VEGF-C, CCL21, EVs	Lymphatic remodeling and transport	Antigen presentation or immune tolerance	Context-dependent

ECM, extracellular matrix; LOX, lysyl oxidase; HA, hyaluronic acid; HIF-1α, hypoxia-inducible factor-1 alpha; TGF-β, transforming growth factor-beta; IFP, interstitial fluid pressure; CCR7, C-C chemokine receptor type 7; CCL21, C-C motif chemokine ligand 21; VEGF-C, vascular endothelial growth factor-C; EVs, extracellular vesicles; TDLN, tumor-draining lymph node.

## 4 Immunomechanics in TDLNs

As the first lymphoid organs exposed to tumor-derived signals, TDLNs serve as a critical interface between the TME and systemic immunity. The mechanobiological alterations discussed above not only influence lymphatic dissemination but also progressively reshape the structural and immunological landscape of TDLNs. Understanding how these changes affect LN function is therefore essential for interpreting the biological rationale of LN de-escalation strategies.

**Figure 3:**
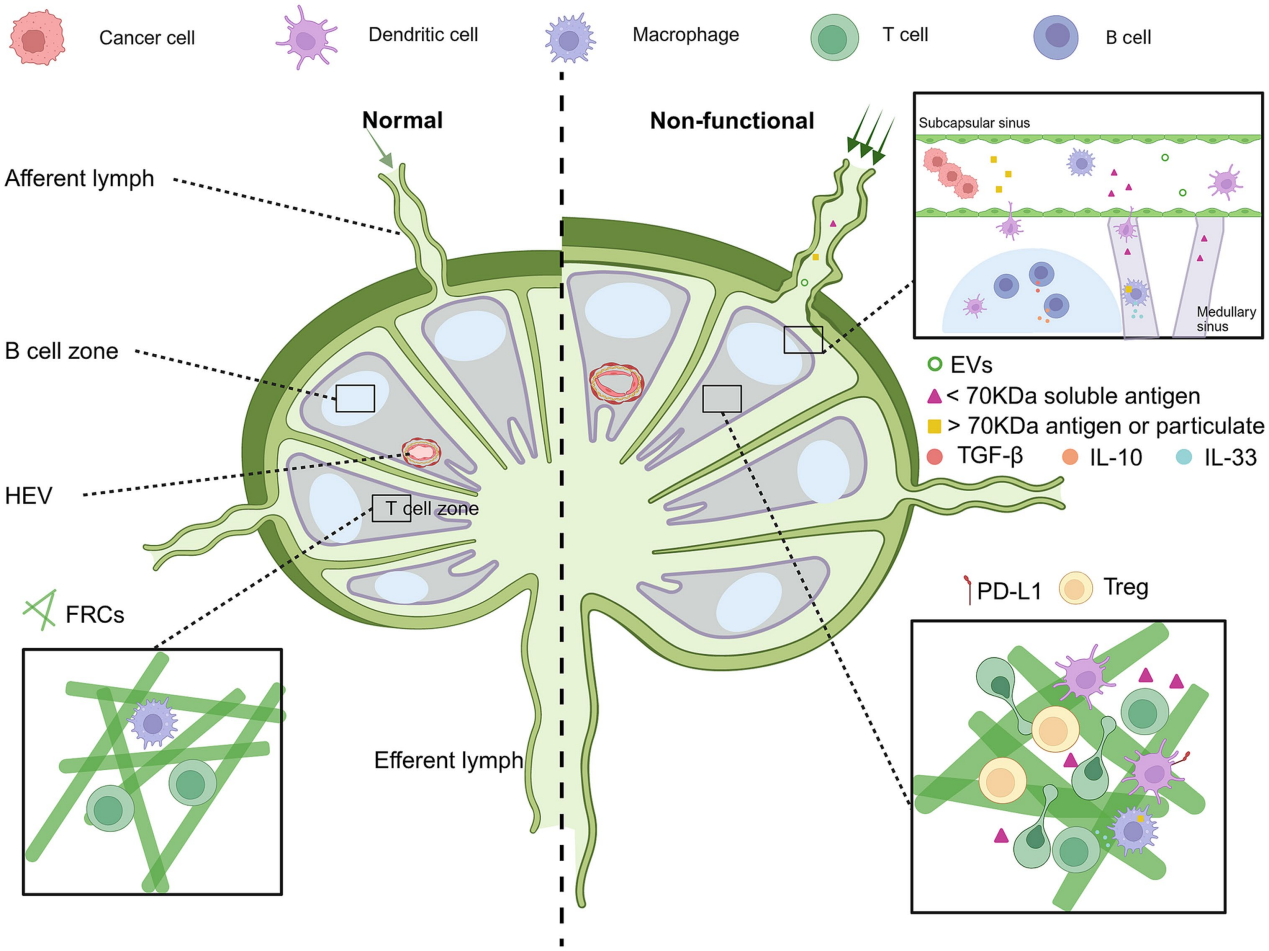
Functional transition of tumor-draining lymph nodes during tumor progression. Under physiological conditions, tumor-draining lymph nodes (TDLNs) maintain a highly organized architecture composed of B-cell follicles, T-cell zones, fibroblastic reticular cells (FRCs), high endothelial venules (HEVs), and lymphatic sinuses, supporting efficient antigen presentation and adaptive immune activation. Soluble antigens and immune cells entering through afferent lymphatic vessels promote dendritic-cell-mediated T-cell priming and antitumor immunity. During tumor progression, persistent exposure to tumor-derived factors, extracellular vesicles (EVs), and lymphatic remodeling drives progressive immunological and structural reprogramming of TDLNs. This process is characterized by accumulation of regulatory T cells (Tregs), increased expression of inhibitory molecules such as PD-L1, production of immunosuppressive cytokines including IL-10 and TGF-*β*, alterations in stromal-cell networks, and impairment of normal lymphocyte trafficking. Recent evidence also suggests that tumor-derived debris may promote immune tolerance through macrophage-mediated IL-33 signaling. Collectively, these changes contribute to the transition from an immune-reactive lymph node to an immunosuppressive and metastasis-permissive state. FRC, fibroblastic reticular cell; HEV, high endothelial venule; EV, extracellular vesicle; Treg, regulatory T cell; PD-L1, programmed death ligand-1. Created in BioRender. Chen, G. (2026) https://BioRender.com/kfaokzo.

Under physiological conditions, LNs are organized into the cortex, paracortex, and medulla (64). The cortex contains lymphoid follicles enriched in B cells, whereas the paracortex is predominantly composed of T cells and also contains HEVs and FRCs (Figure 3). The medulla is formed by medullary cords and sinuses, where natural killer (NK) cells, macrophages, plasma cells, and other immune populations reside. From a structural perspective, the LN can also be broadly understood as consisting of a “supportive phase” and an “primary phase” (65): the former is mainly composed of LN stromal elements, including LECs, HEVs, and FRCs, whereas the latter contains diverse innate and adaptive immune cells. Together, these components make the LN a highly organized site in which innate and adaptive immunity are coordinated to initiate and amplify immune responses.

TDLNs represent the earliest and most direct interface between the local TME and the systemic immune system. Remodeling of TDLN often precedes overt metastasis and is characterized by sinus expansion, restructuring of lymphatic endothelial and stromal cell networks, redistribution of immune cell subsets, and alterations in chemokine programs, all of which together provide the histologic basis of a premetastatic niche (1, 66). Recent work has further suggested that tumor-driven lymphangiogenesis and abnormal lymph flow not only facilitate tumor cell entry into the lymphatic system, but also initiate immunosuppressive programming within the LN before frank metastatic colonization becomes apparent (66, 67).

At the immunological level, TDLNs display a clear duality. In the early stage, an intact LN architecture supports the delivery of tumor antigens to the subcapsular sinus and paracortical regions, where resident and migratory DC coordinate antigen presentation and prime tumor-specific T-cell responses (63, 68). In a human study of HNSCC, Rahim et al. showed that uninvolved regional LN contain abundant progenitor exhausted-like CD8 + T cells that are clonally related to terminally exhausted intratumoral T cells and exhibit activation and differentiation after anti-PD-L1 therapy (63). By contrast, once LN are overtaken by metastasis, this reactive cellular ecosystem is markedly impaired, suggesting that metastatic LN may weaken rather than enhance the response to immunotherapy.

Conversely, TDLNs are also major sites in which immune tolerance is progressively established. This transition is accompanied by both immune-cell reprogramming and stromal remodeling. In metastatic LN, B-cell responses may acquire regulatory features, including the production of IL-10 and TGF-beta, which can suppress effector T-cell activity and promote the expansion of Treg (69, 70). Within the T-cell zone, CD8 + T cells frequently exhibit features of functional exhaustion, while Treg accumulate and helper T-cell (Th) polarization may shift away from Th1-dominant immunity toward a relatively more immunosuppressive state (71, 72). In addition, macrophages in the nodal sinus, including subcapsular and medullary sinus macrophage populations, may contribute to local immune tolerance (62, 73). Another, the stromal compartment is also profoundly remodeled during this process. HEVs may decrease in density and undergo structural alteration, potentially impairing normal lymphocyte trafficking (74). At the same time, FRCs can deposit ECM components such as collagen and fibronectin, creating a denser stromal network that restricts immune-cell migration and reshapes intranodal mechanics (75, 76). FRCs may also alter the secretion of survival and trafficking signals, including IL-7 and CCL21, thereby influencing T-cell maintenance, positioning, and function (77, 78). LEC can contribute to T-cell tolerance through antigen presentation and PD-L1-mediated inhibitory signaling, while Treg, suppressive DCs, and distinct macrophage populations accumulate under persistent tumor-derived lymphatic stimulation (79–82). Accordingly, the LN may function either as an initiator of antitumor immunity or as an amplifier of immune escape and treatment resistance, and its biological role appears to be both time-dependent and plastic (1, 68, 82).

Beyond immune suppression, the LN itself also undergoes substantial biomechanical remodeling. Mechanobiological studies indicate that under inflammatory or tumor-draining conditions, LN volume, stromal tension, cell migration patterns, and vascular and lymphatic organization change in a coordinated manner; excessive or dysregulated mechanical remodeling may shift the node from an efficient immune-reactive state toward functional impairment (77, 83, 84). This framework provides a plausible explanation for why preservation of some LN may be beneficial at certain stages of disease, whereas at other time points the same nodes may instead become permissive sites for tumor progression.

## 5 Perspectives for LN management

If LN are understood as functional nodes within a tumor-immune-mechanics network, de-escalation in surgery or radiotherapy should not rely solely on anatomical distribution, but should increasingly incorporate functional assessment. A key question is which LN are still serving predominantly as sites of antigen presentation and immune amplification, and which have already been remodeled into immunosuppressive or metastasis-promoting niches (63, 68, 79). Equally important is the recognition that the role of the LN may change over the course of treatment, including before and after neoadjuvant immunotherapy, or during different phases of radiotherapy and systemic therapy (68, 85). In parallel, LN-related morbidity should be treated as a clinically meaningful endpoint, rather than continuing high-intensity local treatment when evidence for survival benefit remains limited.

From an operational perspective, future precision LN management will likely depend on integrating multiple dimensions of information, including SLN tumor burden, lymphatic flow and lymphangiogenesis, nodal architectural and mechanical changes, immune-cell composition, treatment-induced dynamic changes, and systemic minimal residual disease signals such as ctDNA (67, 83, 85). In particular, biomechanical parameters, such as peritumoral interstitial flow, ECM stiffness, and the degree of nodal remodeling, may serve as useful bridges linking local disease status to systemic immune activity and may help identify patients who are genuinely suitable for reduced dissection or reduced nodal irradiation (67, 77, 84).

Preoperatively, radiomics- and artificial intelligence (AI)-based imaging approaches may improve nodal risk stratification and help refine the selection of patients for nodal preservation, although these methods remain adjunctive rather than definitive (86). Intraoperatively, molecular-targeted fluorescence imaging and next-generation probes may enhance the detection of occult or micro metastatic nodal disease and thereby support more selective removal of truly involved nodes while sparing clinically negative nodes whenever appropriate (87). More speculatively, artificial or biomimetic LN platforms are being explored in preclinical models as a means of restoring or augmenting immune activation after surgery, but these strategies are not yet ready for routine clinical use (88, 89).

Importantly, de-escalation does not imply abandoning the value of LN management altogether. In patients with high-volume nodal disease, gross extra-nodal extension, a strong need for precise pathological staging to guide subsequent therapy, or a high risk of locoregional failure, active nodal treatment remains necessary. What should be advocated instead is a strategy of selective preservation and selective intensification, bounded by disease-specific evidence, centered on patient benefit, and guided by functional stratification rather than a one-size-fits-all approach.

## 6 Conclusion

Advances in tumor mechanobiology are reshaping the traditional understanding of LN. LN are neither merely anatomical stations nor fixed structures that should always be preserved or removed. Rather, they are dynamic functional organs that continuously change with tumor progression, alterations in lymphatic flow, and immune remodeling. From this perspective, the true significance of LN de-escalation lies not simply in doing less, but in applying more precise criteria to determine which interventions remain necessary and which may be safely avoided.

Taken together, current evidence indicates that MM and BC have already provided a relatively mature clinical foundation for LN de-escalation. In HNSCC, LC, and treatment settings involving radiotherapy or immunotherapy, emerging data suggest that preserving functional TDLN may carry additional therapeutic value. By contrast, in SC and CC, de-escalation strategies still require stronger support from prospective studies. Looking forward, with the continued integration of mechanical phenotypes, immune profiling, and minimal residual disease assessment, LN management may progressively evolve from a traditional anatomy-based approach toward a more functional and individualized strategy, thereby offering new theoretical and practical avenues for precision oncology.

## Glossary

LN: Lymph node
SLNB: Sentinel LN biopsy
ICB: Immune checkpoint blockade
LEC: Lymphatic endothelial cell
Treg: Regulatory T cell
ECM: Extracellular matrix
SS: Solid stress
IFP: Interstitial fluid pressure
TDLN: Tumor-draining LN
SLN: Sentinel LN
BC: Breast cancer
MM: Melanoma
HNSCC: Head and neck squamous cell carcinoma
LC: Lung cancer
SC: Stomach cancer
CC: Colon cancer
CLND: Complete lymph node dissection
ALND: Axillary lymph node dissection
NSCLC: Non-small cell lung cancer
MLN: Mediastinal lymph node
CLN: Cervical lymph nodes
OSCC: Oral squamous cell carcinoma
TME: Tumor microenvironment
DC: Dendritic cell
CAF: Cancer-associated fibroblast
HA: Hyaluronic acid
LOX: Lysyl oxidase
3D: Three-dimensional
TACS: Tumor-associated collagen signatures
EV: Extracellular vesicle
MSM: Medullary sinus macrophage
HEV: High endothelial venule
FRC: Fibroblastic reticular cell
NK: Natural killer
Th: Helper T-cell
AI: Artificial intelligence
